# Bovine serum albumin detection by using molecularly imprinted surface plasmon resonance sensors

**DOI:** 10.55730/1300-0527.3322

**Published:** 2021-12-06

**Authors:** Ali ARAZ

**Affiliations:** 1 Hacettepe University, Department of Chemistry, Ankara, Turkey; 2 Dokuz Eylül University, Department of Chemistry, İzmir, Turkey

**Keywords:** Bovine serum albumin, surface plasmon resonance, sensor, molecularly imprinting

## Abstract

Molecular imprinted polymers (MIP) have key-lock pattern binding properties specific to the size and shape of target molecules. In this study, we have prepared detection platforms based on a molecularly imprinted surface plasmon resonance (SPR) sensor that can detect bovine serum albumin (BSA) sensitively, selectively, quickly, and in real time. The polymeric film prepared on the SPR sensor surface by molecular imprinting method was obtained by selecting the N-methacryloyl-(L)-glutamic acid molecule as a suitable functional monomer using ultraviolet polymerization. Three different imprinting methods, such as epitope, bulk, and surface imprinting methods, were used to examine the imprinting efficiency. Real-time measurements were performed with BSA imprinted SPR sensor provide linearity in the concentration range from 0.10 to 7.50 nM and indicate a detection limit value of 0.015 nM. Furthermore, we performed the selectivity experiments, where transferrin and hemoglobin were chosen as competitor agents. Overall, the SPR sensor prepared by the epitope imprinting approach has been found to be highly selective and sensitive for bovine serum albumin. To statistically assess the reusability of the sensor, intraday experiments were tested three times with five replicates. The RSD% value less than <1.3 indicates high reproducibility for both sensor production and reproducibility of the method. Validation studies were carried out via enzyme-linked immunosorbent analysis technique (ELISA) in order to demonstrate the applicability of the BSA imprinted SPR sensor. Due to their features such as reusability, fast response time, and ease of use, these SPR sensors, which could be used as an alternative to albumin monitoring approaches, can also be adapted to detect and monitor other proteins in real time.

## 1. Introduction

Molecular imprinting technique provides the selectivity of recognition site with ligand selectors in synthetic polymers. For that, atoms, ions, molecules, complexes or micro-organisms can be used as template. Subsequent removal of the template is necessary for molecules to be recognized in spaces emptied by template molecules [1–3]. In recent years, the design, preparation, characterization, and continuous development of molecular imprinted polymers (MIP) have reflected gradual maturation [4–7]. MIPs differ from other detection systems in matters such as their wide application range, high recognition specificity, and structure control. Having advantages such as easy preparation, robust structure and stability, MIPs are impressive in the field of bio-detection, especially artificial antibodies and drug distribution [8,9]. Molecular imprinting technique applied to the sensor surface is divided into three main principles: bulk printing, epitope printing, and surface printing [10]. Since 1990s, the affinity and kinetic properties of protein-protein [11], DNA [12], enzyme-substrate [13], receptor-drug [14], and lipid membrane-protein [15] are being investigated using surface plasmon resonance (SPR) systems.

It offers a very powerful and specific method during the binding of many macromolecules such as polysaccharide [16], cell or virus-protein [17]. SPR sensors measure this precise recognition based on the change in the refractive index that occurs between thin metal layers and the glass prism. Detection of an analyte on the SPR surface is carried out after biorecognition agents have been fixed to the surface. Calculations are made by measuring the change in reflection angle and light intensity expected to occur. With SPR sensors based on this logic, a lot of research has already been done, and it has been proven that it is open to development, and SPR technology has started to be preferred, especially in biomedical, environmental, and industrial areas [18–22]. Examination of many chemical and biological species is available with various SPR measurement systems [23,24].

The most important advantages of SPR sensors are high sensitivity, simple sample preparation, low cost, rapid measurement, and their repeatability. In addition, one of the most important problems encountered in SPR sensors is that molecules with low stability and especially biological properties are denatured during the regeneration stage. In this study, BSA imprinted polymeric film was synthesized using the molecular imprinting method. One of the most important features of this method is that the molecularly imprinted polymers have a stable structure and can be used repeatedly, so they are resistant to harsh environmental conditions.

There are 583 amino acid residues in the blood. One of them, Albumin, is responsible for maintaining the homeostasis of the blood and carrying out small molecules [25]. Bovine serum albumin (BSA), which is widely used in biochemical tests, is a frequently studied blood protein [26–28]. In addition to these functions, it is also used as a blocking agent in polymerase chain reaction (PCR) amplifications. Some methods have been used for BSA detection such as fourier-transform infrared spectroscopy, fluorimetric detecti,on and high performance liquid chromatography (HPLC) [29–31]. High-accuracy results can be obtained with these methods, but the procedures require a lot of preprocessing. Surface plasmon resonance (SPR) is a system that is used for detection based on the refraction caused by the interaction on the metal surface.

Bovine serum albumin (BSA) has a single chain of amino acid residues and 76% similarity to human serum albumin (HSA). BSA, which has an isoelectric point of around 4.7–5.2, is also a very large protein, weighing 66 kDa. There are many studies in the literature about BSA due to its presence in high amounts in blood plasma [32]. It is a suitable protein to be widely studied thanks to its low cost, stable structure, and similarity with HSA [33–35].

SPR sensors are an excellent system that can be used in the detection of biomolecules, since they have many advantages such as easy use, high sensitivity, and not requiring labeling. In this study, studies using BSA imprinted epitope, bulk, and surface imprinting branches of SPR sensors are included. N-methacryloyl-(L)-glutamic acid (MAGA) was chosen as a suitable functional monomer and polymerized with 2-hydroxyethyl methacrylate (HEMA) in the presence of ethylene glycol dimethacrylate (EGDMA) used as crosslinker. Glutamic acid molecule was used for the epitope imprinting technique. BSA imprinted poly(ethylene glycol dimethacrylate-N-methacryloyl-(L)-glutamic acid) [poly(EGDMA-MAGA)] polymeric films are attached to the gold surfaces of the SPR chip. BSA imprinted and non-imprinted SPR chip surfaces were characterized by various techniques such as atomic force microscopy (AFM), ellipsometer, and contact angle measurements. The binding kinetics and detection dynamics of BSA molecules were studied by binding BSA at different concentrations applied to BSA imprinted and non-imprinted SPR sensors. The selectivity, specificity, and reusability features of the BSA imprinted SPR sensor was investigated. It has been found that BSA imprinted polymeric films show greater sensitivity to the target molecule than non-imprinted ones.

## 2. Experimental

### 2.1. Materials

Bovine serum albumin (BSA), 2-hydroxyethyl methacrylate (HEMA), allymercaptane, sodium hydroxide, hydrochloric acid, 3-amino-propyltriethoxysilane (APTES), glutamic acid, glutaraldehyde, α,α′-azoisobutyronitrile (AIBN), and ethylene glycol dimethacrylate (EGDMA) were obtained from Sigma–Aldrich Corporation (MO, USA). Artificial human plasma was supplied by Tokra Medical (Ankara, Turkey). Gold surface chips used in the SPRimager II device were purchased from GWC Technologies (WI, USA).

### 2.2. Preparation of SPR sensors by molecular imprinting method

N-Methacryloyl-(L)-glutamic acid (MAGA) was used as a functional monomer. The synthesis procedure of MAGA functional monomer was synthesized and reported by Denizli et al. [35]. The gold surface of the SPR chip was prepared in three different ways using the molecular imprinting method; for epitope imprinting approach*:* (i) the gold surfaces of SPR chips were modified with allyl mercaptan, (ii) to produce protein stamp, firstly glass slide surface was modified with APTES and then functionalized with glutaraldehyde to give reaction with glutamic acid chosen as epitope part, for epitope approach, (iii) the prepared monomer mixtures were dropped on the gold surfaces of SPR chips modified with allyl groups, (iV) functionalized glass slide was pressed onto the gold chip surface poured monomer mixture, and polymerization processes were initiated in order to convert the monomer mixture into polymeric films with UV light, (V) after polymerization, the slide was removed to make cavities for template molecules for bulk imprinting approach, the steps of which are given as follows: (i) firstly, the SPR chip’s gold surfaces were modified with allyl mercaptan, (ii) bovine serum albumin (BSA), as a template molecule was coordinated with N-Methacryloyl-(L)-glutamic acid (MAGA) monomer to form BSA:MAGA pre-polymerization complex, and then pre-polymerization complex BSA:MAGA was mixed with HEMA monomer to obtain relatively hydrophilic monomer mixture, (iii) after monomer mixtures were dropped on the gold surfaces of SPR chips modified with allyl groups, polymerization processes were initiated with UV light in order to convert the monomer mixtures into polymeric films, (iV) template molecule was removed from the matrice with desorption agent. For surface imprinting approach, (i) the gold surfaces of SPR chips were modified with allyl mercaptan, (ii) to produce protein stamp, firstly glass slide surface was modified with APTES and then functionalized with glutaraldehyde to give reaction with BSA chosen as template for imprinting, (iii) monomer mixtures were dropped on the modified gold surfaces of SPR chips that had allyl groups, (iV) functionalized glass slide was pressed on the gold chip surface poured monomer mixture and UV light has been used to convert monomer mixtures into polymeric films, (V) after polymerization, slide was removed to make cavities for template molecules.

**Figure 1:**
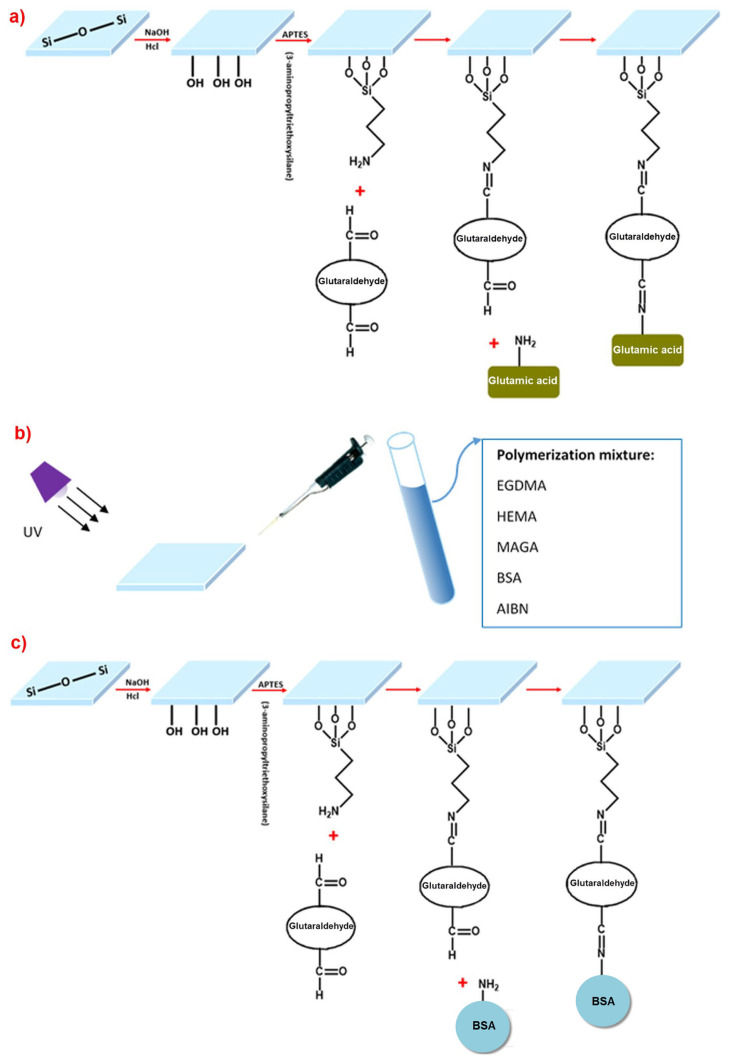
Schematic representation of imprinting types for BSA detection. (A) Preparation glass cover slips (protein stamps) for epitope approach, (B) preparation of SPR chips by bulk polymerization, (C) surface imprinting of BSA on the SPR sensor chip surface via microcontact method.

The SPR chip was incubated in a solution of 3.0 mM allyl mercaptan at 25 °C for 12 h. Then, it was washed with ethanol and dried. The monomer solution was prepared by dissolving the initiator (AIBN) in the monomer mixture. Then, 5 μL of the prepared monomer solution was dropped on the gold surface of the SPR chip and stirred with the spin coater for 5 s to obtain homogeneous polymeric film. Polymerization was initiated with UV lamp (100 W, 365 nm) and continued for 3h. Template removal was performed with acetate buffer containing 4 mM SDS solution for bulk imprinting approach. Specifically, the BSA imprinted SPR sensor was immersed ina desorption solution. The BSA imprinted SPR sensor was dried after washing with ultrapure water and removing the BSA. Epitope, bulk and surface imprinted SPR sensor chip was prepared as shown schematically in Figure 1. The non-imprinted SPR sensor was performed according to the same recipe without using template molecules. Glutamic acid and BSA amount were taken same (50 *μ*mol) with the amount of functional MAGA monomer for polymerization.

### 2.3. Surface characterization of the molecularly imprinted SPR sensor

The surfaces of unmodified and BSA imprinted SPR chips were characterized in tapping mode using an atomic force microscope (AFM) (Nanomagnetics Instruments, Oxford, UK) capable of high-resolution measurements due to the cantilever interferometer. Double-sided carbon strips were used to attach SPR chips to a sample holder in the AFM. Parameters such as oscillation frequency, vibration, and free vibration amplitude affect the result of experimental measurements.

An ellipsometer having auto-nulling imaging properties (Nanofilm EP3, Germany) was used for ellipsometer measurements. After applying the four-zone auto-nulling procedure integrating the sample areas, the algorithm for layer thickness analysis was fitted.

Contact angle (CA) measurements of unmodified, non-imprinted, and BSA imprinted SPR chip surfaces were carried out with the KRUSS DSA100 (Hamburg, Germany) instrument. CA values of SPR chip surfaces were measured using the sessile drop method, and the results were calculated as the average of the contact angles of different drops.

### 2.4. Kinetic studies

Kinetic analysis of BSA imprinted (MIP) and non-imprinted (NIP) SPR sensors was performed with SPRimager II (GWC Technologies, WI, USA). Firstly, to wash the BSA imprinted and non-imprinted SPR sensors, 50 mM NaOH solution and deionized water (50 mL, 150 μL/min) were applied for 10 min. The sensor surfaces were then equilibrated with adsorption buffer (pH = 4.0, acetate buffer) for 30 min. Kinetic studies were performed at the resonance angle set with the mirror system. Buffer circulation was continued for several minutes using the SPR system. Sample solutions prepared to contain BSA were applied to the SPR system (20 mL, 150 μL/min). The change in reflection values (%DR) was monitored, and it was determined that a plateau reached within 5 min. The desorption process was done by applying pH = 4.0, acetate buffer containing 4 mM SDS solution (20 mL, 150 μL/min). The BSA imprinted SPR sensor was washed with ultrapure water followed by pH = 4.0 acetate buffer containing 4 mM SDS solution afterward. BSA solutions within the range of 0.10–7.50 nM were applied to the SPR system to determine the concentration dependence of the BSA imprinted SPR sensor response.

Hemoglobin (Hb) and transferrin (Tf) proteins were used as competing molecules to study the selectivity of BSA imprinted SPR sensors. The solutions of competing molecules were prepared with the same concentration (7.5 nM), and their interactions with BSA imprinted and non-imprinted SPR sensors were examined separately to compare the selectivity behavior. Selectivity (k) and relative selectivity (k’) coefficients were calculated according to kinetic analysis of BSA imprinted and non-imprinted SPR sensors. The values k and k’ are described by the following equations [37,38].


(6) $$\text{k}=\Delta {\text{R}}_{\text{template}}/\Delta {\text{R}}_{\text{competitor}}$$

(7) $${\text{k}}^{\prime }={\text{k}}_{\text{imprinted}}/{\text{k}}_{\text{non-imprinted}}$$

The reproducibility of BSA imprinted SPR sensor was tested for detection of BSA from artificial plasma. Artificial plasma was diluted with adsorption buffer (pH = 4.0, acetate buffer) in the ratio of 1/2 (v/v). The aqueous solutions of BSA at concentration of 2.5 nM and 5 nM were spiked in the diluted artificial plasma 1/2 (v/v). BSA imprinted SPR sensor was equilibrated with adsorption buffer (pH = 4.0, acetate buffer). Then, the spiked BSA artificial plasma samples were applied to the SPRimager II system. The removal of BSA molecules on the surface of BSA imprinted SPR sensor was carried out with a pH value of 4.0 acetate buffer containing 4 mM SDS solution. Kinetic analysis of BSA imprinted SPR sensor was compared with the results obtained from ELISA measurements.

## 3. Results and discussion

### 3.1. Characterization studies

Characterization of SPR chip surfaces was performed by atomic force microscope (AFM), ellipsometry, and contact angle measurements.

**Figure 2:**
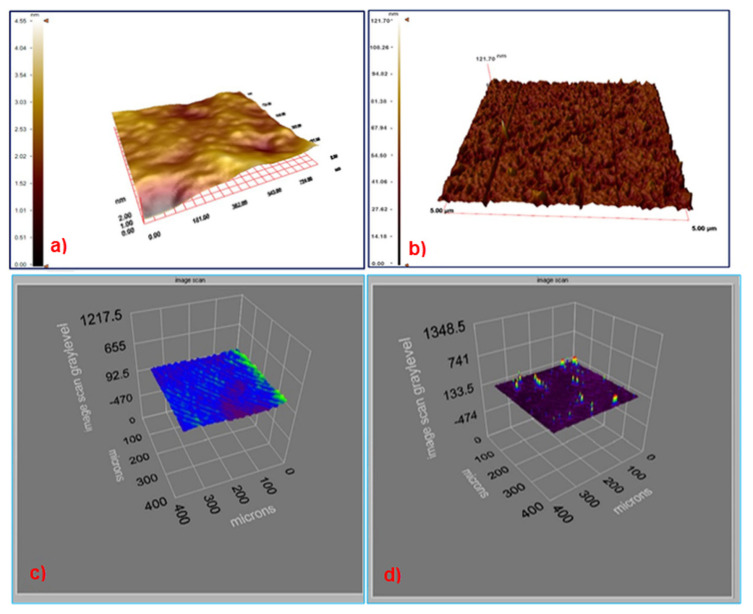
The AFM images (a. unmodified and b. BSA imprinted SPR chip surfaces). Ellipsometry images (c. unmodified and d. BSA imprinted SPR chip surfaces).

The AFM images of unmodified and BSA imprinted SPR chip surfaces were shown in Figures 2A and 2B. The polymerization process made the surface of BSA imprinted SPR chip surfaces rough. Polymeric film formation is homogeneous according to AFM images. The surface deepness for unmodified and BSA imprinted SPR chip surfaces were 4.55 ± 0.3 nm and 121.7 ± 0.1 nm, respectively. Ellipsometry measurements were made to determine the thickness of the SPR chip surfaces after the molecular imprinting process. According to the results, the thicknesses of unmodified and BSA imprinted SPR chip surfaces were estimated to be 92.5 ± 0.8 nm and 135.5 ± 0.7 nm, respectively (Figures 2C and 2D). These results were consistent with ellipsometry results. AFM and ellipsometry measurement results showed that BSA imprinted SPR chip surfaces have rough surfaces, and the thickness differences of SPR chip surfaces proved that the imprinting process was carried out successfully.

**Figure 3:**
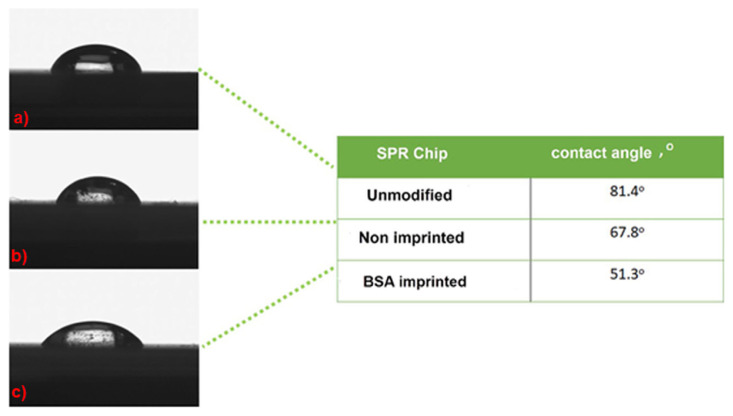
Contact angle images for SPR chip surfaces (a. unmodified SPR chip surface, b. non imprinted SPR chip surface, and c. BSA imprinted SPR chip surface).

Contact angle (CA) measurements for unmodified, BSA imprinted and non-imprinted SPR chip surfaces were also summarized in Figures 3A–3C. The CA value of unmodified SPR chip surfaces was determined to be 81.4°. After the molecular imprinting of BSA, the CA values of BSA imprinted and non-imprinted SPR chip surfaces were determined as 51.3°, and 67.8°, respectively. CA values decreased after the imprinting process took place. The addition of BSA may cause the hydrophilicity and/or polarity of the surface to increase. After preparing the non-imprinted polymeric film, the CA value of the sensor surface decreased from 81.4° to 51.3°. This indicates that the polymeric film formed on the modified SPR sensor has hydrophilic properties resulting from the HEMA monomer.

### 3.2. Kinetic analysis

**Figure 4:**
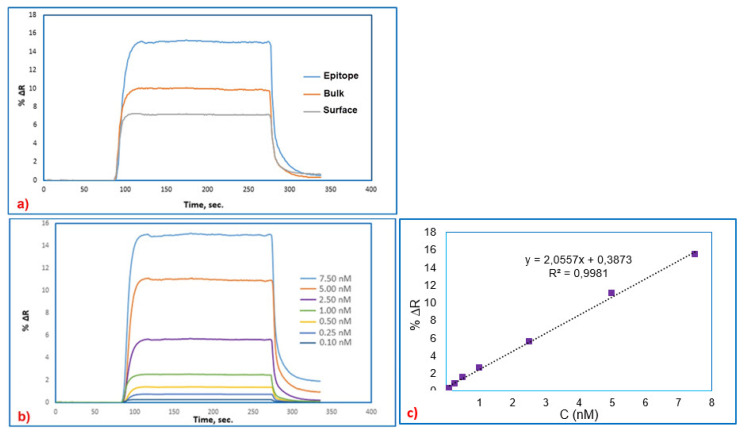
The real-time BSA detection with SPR sensors prepared by different approaches, epitope, bulk, surface (a), the real-time BSA detection at different concentrations, (b) SPR sensor response for the concentration ranges imprinting methods.

To understand which type of imprinting process is superior having good efficiency, SPR sensors obtained by epitope, bulk, and surface imprinting methods were treated with 7.5 nM BSA solution. When the results obtained from the analyses using the SPR system, which was produced with the three different imprinting systems, were compared, higher ΔR result obtained from the epitope approach shows that this SPR system is more sensitive than the other two SPR systems including bulk and surface imprinting system. Figure 4A shows the ΔR results for the three imprinting approaches. When ΔR results were compared, it was decided to continue with the SPR sensor system obtained by epitope approach for future studies.

BSA imprinted SPR sensor prepared by epitope approach was used for real-time detection of BSA from aqueous solutions. For this purpose, BSA imprinted SPR sensors interacted with the aqueous solutions of BSA in the concentration range of 0.10 nM to 7.50 nM. As seen in Figure 4B, BSA imprinted SPR sensor had quick responses when the BSA solutions reached the SPR sensor surfaces. The acetate buffer having a pH value of 4.0 and containing 4 mM SDS solution was applied to BSA imprinted SPR sensors as desorption agent, and then ultrapure water passed through the SPR system before injecting BSA solutions. After that, to the SPR system, solutions containing different BSA concentrations were injected. As expected, increasing the BSA concentration resulted in an increase in BSA imprinted SPR sensor response. The desorption agent was injected into the SPR sensor after plateau values were reached within 5 min. In the final stage, the system was washed by passing ultrapure water through the SPR sensor system for 3 min.

As shown in Figure 4, increasing concentrations of BSA solutions resulted in increased SPR responses. The change in reflectance (ΔR) increased in proportion to the ratio of increase with an increase in concentration from 0.10 nM to 7.5 nM. The relationship between the concentration of BSA solutions and %ΔR has proven that BSA imprinted SPR sensor response is linear. In this study, the linear equation in the linear range of 0.1–7.50 nM concentration is y = 2.0117x+0.379 with a coefficient of 0.996. Using the kinetic data of the f BSA imprinted SPR sensor, the limit of detection (LOD=3.3 S/m) was calculated from the slope of the calibration curve of BSA molecules. The standard deviation of the intercept is S, and the slope of the regression line is m [18,22]. The estimated LOD value was recorded as 0.015 nM for BSA imprinted SPR sensors.

For the preparation of high selectivity, real-time, low-cost, reusable SPR sensors for BSA detection, BSA-imprinted SPR sensors have been prepared by using the advantages of both sensor systems and molecular imprinting method. In this study, the best imprinting efficiency was determined on the sensor surface using 3 different imprinting methods. The total analysis time is about 5.67 min. BSA solutions at different concentrations were observed by performing kinetic analyzes using BSA imprinted SPR sensors, where BSA can be determined from both aqueous solution and artificial plasma. BSA imprinted SPR sensors prepared for BSA detection are compared with the studies in the literature and given in Table 1.

The association kinetics analysis and equilibrium analysis approach (Scatchard) are calculated in Table S1. Langmuir, Freundlich and Langmuir-Freundlich isotherm models have been applied to BSA imprinted SPR sensor in Table S2. The linearity of the Langmuir isotherm model was better than the other two isotherm models. With a linearity of ΔR_max_ of 17.9, the Langmuir model, therefore, optimally explained the interaction between the BSA molecules and BSA imprinted SPR sensor.

### 3.3. Selectivity studies

In order to show the selectivity of the BSA imprinted SPR sensor, structurally related molecules transferrin and hemoglobin were used as competitor agents for each other. Transferrin (Tf, MW: 80 kDa) and Hemoglobin (Hb, MW: 64.58 kDa) as competitor agents, which are close to bovine serum albumin (BSA, MW: 66.5 kDa) in terms of both molecular weight and structure, are used in the selective studies. The selectivity coefficients for BSA imprinted and non-imprinted SPR sensors and the relative selectivity coefficients were given in Table 2. By comparing the relative selectivity coefficients of BSA imprinted SPR sensors (BSA/Tf, and BSA/Hb) as shown in Table 2, it can be deduced that BSA recognition sites have been created during the imprinting process and recognized BSA preferentially. The relative selectivity coefficients of BSA imprinted SPR sensors to non-imprinted SPR sensors for BSA between BSA/Tf and BSA/Hb were determined as 1.59 and 1.22.

In this study, functional monomer MAGA, which is an amino acid-based monomer, assemble around the template BSA molecule by interacting with functional groups on BSA structure, leaving behind a selective binding site after polymerization. The relative selectivity coefficients obtained for the BSA molecule show that the cavities created in BSA imprinted polymeric film recognize imprinted molecules preferentially and have structural memory and amazing molecular size matching with template molecules. The imprinting factor showing the imprinting efficiency was recorded as 4.08 and implied that the imprinting process was accomplished successfully. When the imprinting approach types were evaluated in Table 3, it was deduced that epitope imprinting is more selective and efficient than the other type of imprinting processes namely, bulk and surface imprinting.

### 3.4. Detection of BSA from artificial plasma

**Figure 5:**
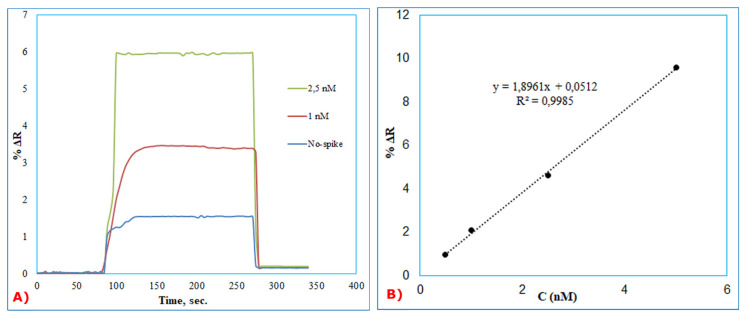
Kinetic analysis for real-time BSA detection (A) and calibration curve (B) of different BSA concentrations (0.5–10 nM) in ½ diluted artificial plasma.

In order to show the reproducibility of BSA imprinted SPR sensors, the aqueous solutions of BSA at a concentration from 1 nM and 2.5 nM were spiked in the diluted artificial plasma 1/2 (*v*/*v*). Firstly, the BSA imprinted SPR sensor was equilibrated with pH = 4.0, acetate buffer. After, artificial plasma without spiked BSA was applied to the SPR system, and then artificial plasma samples spiked with BSA at a concentration of 1 nM and 2.5 nM were applied to the SPRimager II system in Figure 5A. Finally, removal of the bounded BSA molecules on the sensor surface was used with acetate buffer having a pH value of 4.0 and containing 4 mM SDS solution. The refractive index changes (DR) of BSA imprinted SPR sensor was monitored in all kinetic analysis. The linear equation in the linear range of 0.5 to 10 nM concentration was y=1.8961x+ 0.0512 with a coefficient of determination of 0.9985 (Figure 5B). To determine the accuracy of the BSA imprinted SPR sensor, the results of the SPR sensor and ELISA are given comparatively in Table 4. The compatibility of the ELISA results with BSA imprinted SPR sensor results shows that SPR sensor is sensitive, accurate, and quantitative for the detection of BSA in artificial plasma samples.

### 3.5. Reusability studies

**Figure 6:**
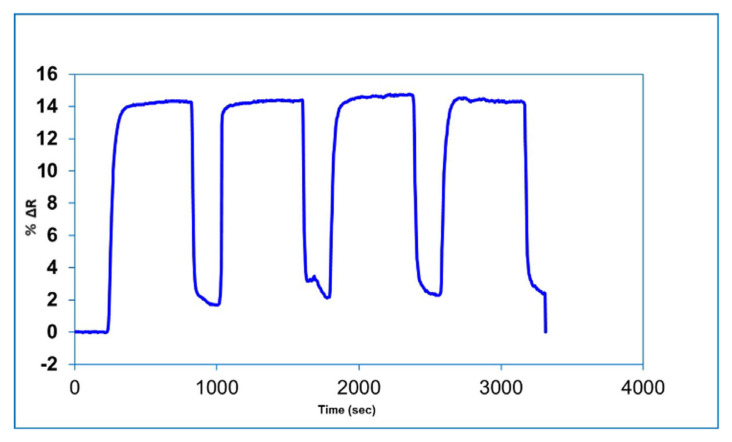
Reusability of BSA imprinted SPR sensor.

One of the major advantages of molecularly imprinted SPR sensors is that they can be reused under long-term storage conditions without any performance loss. Reusability studies of the BSA imprinted SPR sensor has been demonstrated using aqueous BSA solutions with four repeated equilibration-adsorption-desorption-regeneration cycles. For this purpose, 7.5 nM BSA solution interacted sequentially with the BSA imprinted SPR sensors. The reflectivity change values for the obtained four cycles was shown in Figure 6. As can be seen from the figure, the BSA imprinted SPR sensor can be successfully reused with repeatable results.

**Figure 7:**
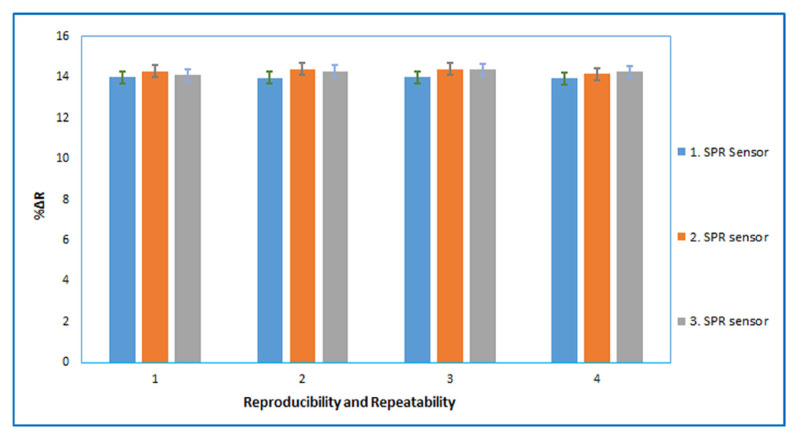
The intraday assays for different BSA imprinted SPR sensors prepared by different batches; three cycle (n = 3) with four replicates for each sensor.

The reproducibility describes the consistency of different sensors prepared under the same set of conditions. We produced three BSA imprinted SPR sensors under the same preparation conditions by different batches. 7.5 nM BSA solution was applied to the prepared sensors, for the intraday assays (three times with four replicates) in Figure 7. The reproducibility of the proposed method has been ascertained by precision studies. The signal intensity repeatability studies of the BSA imprinted SPR sensor was evaluated statistically for the intraday assays (three times with four replicates), and the accuracy of the repeatability was confirmed by calculating the percent relative standard deviation (RSD%). The RSD% value less than <1.3 indicates high reproducibility for both sensor production and reproducibility of the method.

## 4. Conclusion

Both bovine serum albumin and human serum albumin proteins are proteins that serve as transport, found in the blood in an abundant amount, such as 52%–60% [47]. These proteins, which have more than one lipophilic binding site in their sub-areas, can both carry and distribute many biological substances such as vitamins, drugs, and hormones [48–52]. As the amount of binding affinity between these proteins and compounds increases, the physiological activity of various materials such as drugs in blood plasma decreases. Therefore, it is important to investigate the binding affinity between serum albumin and different compounds. The discovery of fast, low-cost, and sensitive applications in biological analysis is a broad research area. SPR sensors, which can incorporate these properties, are suitable to serve the purpose with good reproducibility when combined with molecular imprinting technology. In this study, a new SPR sensor was prepared using the molecular imprinting method for selective recognition of BSA in an aqueous solution. Scatchard, Langmuir, Freundlich, and Langmuir–Freundlich equilibrium and adsorption isotherm models used to determine the kinetic and binding constants of the BSA imprinted SPR sensors implied that Langmuir adsorption isotherm model was best-fitted to the BSA imprinted SPR sensor. Also, the selectivity studies proved that the formed cavities recognized BSA with high selectivity than the other two transferrin and hemoglobin molecules. The BSA imprinted SPR sensor prepared by the epitope approach showed high sensitivity and efficiency. Its superior features, such as easy preparation, cheapness, and reusability make it advantageous. Thus, the SPR sensor could be used for label-free detection and real-time monitoring of BSA. Herein, in the light of obtained data, we have focused on producing the BSA imprinted SPR sensor, which can be used as a potential alternative to detect BSA.

## Supporting File

### 1. Equilibrium and binding analysis

Equilibrium analyzes performed during SPR measurements were evaluated to determine host-guest interaction between BSA solutions and BSA imprinted SPR sensors.

The kinetic and equilibrium isotherm coefficients was calculated by using a pseudo-first-order kinetic analysis, equilibrium binding parameters (Scatchard). The applied linear forms for the models are as follows:


(1) $$\text{Equilibrium kinetic analysis:}\;\text{d}\Delta \text{R}/\text{dt}={\text{k}}_{\text{a}}\text{C}\Delta {\text{R}}_{\text{max}}-({\text{k}}_{\text{a}}\text{C}+{\text{k}}_{\text{d}})\Delta \text{R}$$

(2) $$\text{Scatchard:}\;\Delta {\text{R}}_{\text{ex}}/\text{C}={\text{K}}_{\text{A}}(\Delta {\text{R}}_{\text{max}}-\Delta {\text{R}}_{\text{eq}})$$

The association constant *(*K_A_*)* calculated from the equilibrium and association kinetic analysis results as 0.183 nM^−1^ indicated high-affinity binding for BSA and results were tabulated for BSA imprinted SPR sensors as in Table S1. Figure S1A and Figure S1B show calculation of kinetic rate constants by association kinetics analysis approach and equilibrium analysis approach (Scatchard), respectively. Scatchard model explains surface heterogeneity for BSA imprinted SPR sensors having different binding sites with similar binding affinity to BSA molecules [1,2]. The K_A_ value showing affinity strength for BSA recognition was calculated as 0 0.27 nM^−1^.

### 2. Adsorption isotherms

The adsorption models (such as Langmuir, Freundlich and Langmuir–Freundlich isotherm models) can be used to define recognition ability, interaction selectivity, and surface homogeneity of the molecular imprinting-based sensors. The Langmuir adsorption isotherm model is generally fit to binding isotherms of molecularly imprinted systems [3,4]. However, many studies have shown that most molecularly imprinted polymers have heterogeneous binding sites. The Freundlich adsorption isotherm model, which shows good fit to the molecular imprinting adsorption isotherm data in the low concentration region (nM-mM), generally refers to a heterogeneous model. However, it cannot be used both in modeling saturation behavior and deviates from experimental isotherms in high concentration regions, consequently used to demonstrate multilayer binding of analyte molecules [5]. The Langmuir–Freundlich adsorption isotherm model provides information about the heterogeneity of the adsorption behavior over a wide concentration range.

Adsorption isotherm models (Langmuir, Freundlich, and Langmuir–Freundlich) was applied to the BSA imprinted SPR biosensor and calculated adsorption isotherm model parameters. The applied linear forms for the models are as follows:


(3) $$\text{Langmuir:}\;\Delta \text{R}=\{\Delta {\text{R}}_{\text{max}}[\text{C}]/{\text{K}}_{\text{D}}+[\text{C}]\}$$

(4) $$\text{Freundlich:}\;\Delta \text{R}=\Delta {\text{R}}_{\text{max}}[\text{C}]\;1/\text{n}$$

(5) $$\text{Langmuir}-\text{Freundlich}:\Delta \text{R}=\{\Delta {\text{R}}_{\text{max}}[\text{C}]1/\text{n}/{\text{K}}_{\text{D}}+[\text{C}]1/\text{n}\}$$

Where ΔR is the measured response after bound; C is BSA concentration (nM); 1/n refers Freundlich exponent; k_a_ (nM^−1^s^−1^), and k_d_ (s^−1^) values refer to the kinetic rate constant for the forward and reverse reactions; K_A_ (nM^−1^) and K_D_ (nM) also refer to the equilibrium constants for the forward and reverse reactions; subscripts max, eq and ex indicate maximum, equilibrium and experimental, respectively. k_a_ and k_d_ values were calculated by plotting the slope of the curves vs to the concentrations by applying equilibrium kinetic analysis.

Among the applied adsorption models, since the Langmuir isotherm model has a high regression coefficient, it was determined as the adsorption isotherm model that best fits the adsorption of (R^2^ = 0.9991, ΔR_max_ = 17.09). It is estimated from the results that BSA is bound to the BSA imprinted SPR sensors as a monolayered. As shown in Table S2, the ΔR_max_ values obtained from the Langmuir adsorption isotherm model are closer to the experimental ones. Figure S2 shows adsorption isotherm models for BSA Adsorption: (a) Langmuir, (b) Freundlich, (c) Langmuir–Freundlich adsorption models.

Figure S1 Association kinetic analysis result for BSA imprinted SPR sensors (A) and Determination of kinetic rate constant for BSA with Equilibrium analysis approach (Scatchard) (B).

Figure S2 Adsorption isotherm models for BSA adsorption: (A) Langmuir, (B) Freundlich, (C) Langmuir–Freundlich adsorption models.

**Table S1:** Equilibrium and association kinetic analysis for BSA imprinted SPR sensor.

Equilibrium Analysis (Scathard)		Association Kinetic	
ΔR _max_	18.93	k _a_, nM ^−1^.s ^−1^	0.0572
**K** **_A_** **, nM** **^−1^**	0.27	**k _d_**,s ^−1^	0.3122
**K** **_D_** **, nM**	3.7	**K _A_**, nM ^−1^	0.1832
**R** **^2^**	0.9823	**K _D_**, nM	5.5
		**R** **^2^**	0.8322

**Table S2:** Calculated adsorption isotherm model parameters for the BSA imprinted SPR sensor.

Langmuir		Freundlich		Langmuir–Freundlich	
**ΔR** **_max_**	17.09	**ΔR** **_max_**	2.55	**ΔR** **_max_**	24
**K** **_D_**	5.4	**1/n**	0.888	**1/n**	0.888
**K** **_A_**	0.1827	**R** **^2^**	0.9714	**K** **_D_**	10.11
**R** **^2^**	0.9991			**K** **_A_**	0.1
				**R** **^2^**	0.9983

References1

LinLP
HuangLS
LinCW
LeeCK
ChenJL
HsuSM
LinS

Determination of binding constant of DNA-binding drug to target DNA by surface plasmon resonance biosensor technology, Curr
Drug Targets
5
2005
61
72
10.2174/1568008053174697157772052

ErtürkG
UzunL
TümerMA
SayR
DenizliA

Fab fragments imprinted SPR biosensor for real-time human immunoglobulin G detection, Biosens
Bioelectron
28
2011
97
104
10.1016/j.bios.2011.07.004218029383

KrishnamoorthyG
CarlenET
Van Der BergA
SchasfoortRBM

Surface plasmon resonance imaging based multiplex biosensor: integration of biomolecular screening detection and kinetics estimation, Sensor
Actuators B Chem
148
2010
511
521
4

LiX
HussonSM

Adsorption of dansylated amino acids on molecularly imprinted surfaces: a surface plasmon resonance study, Biosens
Bioelectron
22
2006
336
348
10.1016/j.bios.2006.04.016167532925

UmplebyRJ
BaxterSC
ChenY
ShahRN
ShimizuKD

Characterization of molecularly imprinted polymers with the Langmuir-Freundlich isotherm
Anal Chem
73
2001
4584
4591
11605834
10.1021/ac0105686

## Figures and Tables

**Table 1:** The comparison of the prepared sensor system in the literature for detection of BSA.

Method	Linear Range	LOD	Retention Time	RF
Electrochemical sensor	0.02–10 μmol/L	0.012 μmol/L	-	39
Localized surface plasmon resonance	0–0.1 μg/mL	0.18 ng/mL	-	40
Electrochemical sensor	1.0–150 ng/mL	0.02 ng/mL		41
Biosensor	0–1 mg/mL	2.413 mg/mL		42
Amperometric	1–7 mM of BSA	-	-	43
Optical fiber biosensor	0.2–2 mg/mL	2.57x10^−4^ mg/mL	-	44
SPR biosensor	10–50 μg/mL	0.45 μg/mL	15 min	45
Surface plasmon resonance	0.02–0.8 mg/mL	0.02 mg/mL	~50 min	46
Surface plasmon resonance	0.10–7.50 nM	0.015 nM	~6 min	This work

**Table 2:** The selectivity coefficients for BSA imprinted (MIP) and non-imprinted (NIP) SPR sensors.

Molecules	MIP Sensor		NIP Sensor		
	ΔR	k	ΔR	k	k′
**BSA**	15.00	-	3.68	-	-
**Tf**	3.10	4.19	1.40	2.62	1.59
**Hb**	1.60	9.37	0.48	7.66	1.22

**Table 3:** Different imprinting approaches on the selectivity and efficiency of BSA for BSA imprinted SPR sensor.

Imprinting approaches	BSA	Hb	k	
**Epitope**	15.00	1.6	9.37	More Efficiency More Selectivity
**Bulk**	10	3	3.3	Medium Efficiency Less Selectivity
**Surface**	7	4.1	1.7	Less Efficiency Less Selectivity

**Table 4:** The recoveries of BSA in artificial plasma samples.

Added BSA (nM)	Found BSA (nM)		Recovery (%)	
	SPR sensor	ELISA	SPR sensor	ELISA
1	0.97 ± 0.008	0.95 ± 0.015	97 ± 0.816	95 ± 1.50
2.5	2.47 ± 0.008	2.46 ± 0.010	98.8 ± 0.327	98.6 ± 0.40
